# Assessment of Pharmacists Prescribing Practices in Poland—A Descriptive Study

**DOI:** 10.3390/healthcare9111505

**Published:** 2021-11-05

**Authors:** Agnieszka Zimmermann, Jakub Płaczek, Natalia Wrzosek, Artur Owczarek

**Affiliations:** 1 Department of Medical and Pharmacy Law, Medical University of Gdańsk, 80-210 Gdańsk, Poland; agnieszka.zimmermann@gumed.edu.pl (A.Z.); natalia.wrzosek@gumed.edu.pl (N.W.); 2 Department of Pharmacoeconomics and Pharmacy Law, Collegium Medicum in Bydgoszcz Nicolaus Copernicus University in Torun, 85-089 Bydgoszcz, Poland; jakub.placzek@cm.umk.pl; 3 Department of Drug Form Technology, Wroclaw Medical University, 50-556 Wroclaw, Poland

**Keywords:** prescriptions, community pharmacy services, emergency prescribing, pharmacy practice, pharmacy law and regulation, COVID-19

## Abstract

Pharmacists play a beneficial role in supplying medicines to patients. Pharmacist prescribing practices were introduced into law in Poland in 2002, permitting pharmacists to prescribe medications in emergency situations and in 2020 the new law allowed to prescribe in all situation where it is needed because of the health risks reasons. Our aim was to analyze pharmacist prescribing practices in Poland and confirm the useful of pharmacists’ activity in this area. Additionally, pharmacists were also authorized to issue reimbursed prescriptions for themselves or their family members. Since January 2020, only e-prescriptions are allowed in Poland. A retrospective analysis of the inspection written reports from 842 community pharmacies in the representative region of Poland with a population of two million, carried out in the time period from 2002 to 2016 was performed (2189 prescriptions) to assess the emergency pharmacist prescribing practices in Poland. The second part of the research was based on digital data on pharmacists prescriptions (18,529) provided by the e-Health Centre (a governmental organization under the Ministry of Health responsible for the development of health care information systems in Poland), enabling to conduct the analysis of pharmacist’s prescribing from 1 of April 2020 to 31 of October 2020. The analysis gave the insight of the evolution of the pharmacy prescribing patterns. In general, pharmaceutical prescriptions were issued in cities with more than 100,000 inhabitants, in town- or city center pharmacies, and in pharmacies in residential areas. The most common reason for a pharmaceutical prescription was that the patient was running out of a medicine and was unable to contact their physician. Cardiovascular, respiratory, dermatological, and digestive medications were most frequently prescribed. An analysis of pharmacists’ prescribing data from 1 April 2020 to 31 October 2020 confirmed the rapid increase of pharmaceutical prescriptions following implementation of the new legislative act during the COVID-19 epidemic.

## 1. Introduction

In its classic sense, a prescription is a specific message conveyed by a person authorized to issue such a document to a person who fills it in a pharmacy. It contains information on the medicines prescribed to the patient, data identifying both the patient and the author of the prescription, as well as the place and date of issue. In Poland, the right to prescribe belongs to physicians, nurses, midwifes and, pharmacists [1].

Pharmacist prescribing was legislated in Poland on 1 October 2002. It was limited only to emergency situations. Prior to this date, medical dispensing was determined by the type of medicine. A professional pharmacy employee (either the pharmacist or the pharmacy technician) was permitted to dispense a single packet of a medicine, which was reserved for medical prescribing, provided it was allocated to an adequate dispensing category, i.e., Rp. (Rp with a dot). However, patients have to pay 100% costs of the drug. Pharmacist emergency prescribing was possible only if “immediate risk to patient health” appeared. However, in Polish Pharmaceutical Law this term was inadequately defined. Instead, a de facto definition, derived from other regulations governing medical emergency services, became commonplace based on the assumption that “immediate risk” means the sudden onset of symptoms associated with serious damage to bodily functions, bodily injury or death, which may require an immediate or emergency medical response. Polish case law also added additional context to a health hazard, which included the need to demonstrate an imminent threat and therefore the significant probability of personal injury and risk of mortality, whether immediate or impending. In the pharmacy, the pharmacist assesses each case and determines whether there is an imminent health risk. The problem of defining what an immediate need is in pharmaceutical practice is also reviewed in the literature [2]. “Emergency prescribing” in exceptional cases, for instance, when there is an immediate risk to patient health, was introduced in several countries including the UK, Australia, the United States, and Canada [3,4]. In the UK, as in Poland, a pharmacist has discretionary power to dispense prescription-only medicine when a patient is unable to present the appropriate prescription and after interviewing the patient. This commonly occurs when a travelling patient forgot to pack their medication. Psychotropic medications and narcotics, except for phenobarbital (limited to a five-day course of treatment) for epilepsy sufferers, are excluded from these provisions. All such dispensing requires formal documentation [4] including a description of the exceptional circumstances. The pharmacist’s discretion only extends to dispensing medicine and to the continuation of existing therapy, but does not authorize the pharmacist to initiate new therapy.

Beginning on 1 April 2020, during coronavirus epidemy, pharmacists were permitted to issue a prescription to any patient in any situation that posed a risk to health. Pharmacists may exceed the 180 days prescribing limit for consecutive pharmacist-prescribed renewals in circumstances where the patient is unable to be assessed by their primary care provider. This temporary provision allows pharmacists to exceed the 180 days prescribing limit for renewals during a public health emergency/crisis for patients who do not have a primary care provider or are unable to access them at this time. Pharmaceutical prescriptions may be issued for all medicines except for narcotics and psychiatric medications. The patient does not receive any reimbursement from the NHF. The pharmacist is not, however, authorized to carry out physical examinations, but must perform a diagnostic interview. Since 1 April 2020, the pharmacist was also authorized to self-prescribe. They are allowed to issue a reimbursed prescription for themselves or their family members. Initial statutory provisions (effective from 2002 to January 2020) required a pharmacist to issue a paper version of the prescription, but this was replaced by electronic documentation since January 2020. The record of each pharmaceutical prescription can identify the person who entered data and the date the prescription was filled. Table 1 summarizes Polish policies for issuing prescriptions by the pharmacist before and after the COVID-19 epidemic.

Pharmacists could prescribe in minor ailments. Enabling pharmacist prescribing for minor ailments to make easier access to health care in a more timely fashion, help improve health outcomes, and reduce costs-per-visit for a variety of non-critical medical conditions. This demonstrates that a significant number of patient visits to walk-in clinics, family doctors, and emergency departments would be prevented, as patients could receive care at the pharmacy. Minor ailments are considered health conditions that can typically be self-diagnosed by patients such as urinary tract infections, upper respiratory tract infections, contact dermatitis, conjunctivitis, and athlete’s foot and can be managed with minimal treatment or straightforward self-care strategies [6]. Pharmacists could also prescribe in chronic conditions mainly renewals prescriptions. Chronic conditions are the largest cause of death and disability in the world. In ambulatory care settings, pharmacists assume responsibility for the management of chronic conditions such as hypertension, asthma, diabetes, hyperlipidaemia, and psychiatric disorders [7].

To date, no study has been published that comprehensively describes pharmaceutical services associated with pharmacist prescribing in community pharmacies in Poland. The primary purpose of reviewing pharmacist prescribing practices, which is still evolving in Poland, is to help fill this knowledge gap. As of the 1 April 2020, Poland a country in Central Europe with a population of 37 million, has substantially expanded the pharmacists’ competences by implementing the regulation that permits to issue a prescription to any patient in any situation posing a risk to health. This regulation and its consequences for the healthcare system may in the close future have significant impact on pharmaceutical regulation in other countries of the Central and Eastern Europe region, where Poland is the only one which permits pharmacists to prescribe [8,9].

The objective of this study was to analyze pharmacist prescribing practices in the Polish healthcare system and describe the evolution of that pharmacist service during 18 years. Comparison of prescribing practices including the most common therapeutic areas was made to find evidence for an expansion in the scope of pharmacists’ practice in Poland. Findings may give an insight for future service planning in other European countries with no pharmacy prescribing service existing. Findings of this research add to the international literature on pharmacist prescribing and through identification of models of prescribing and medicines prescribed to inform future education and policy.

## 2. Materials and Methods

### 2.1. Study Designed and Settings

To understand and find the scope of pharmacist prescribing in Poland, the researchers conducted an analysis of the emergency prescription conditions and practice and then compared this data with an analysis of the actual patterns of prescribing by pharmacist prescribers. Data were obtained from two sources. A retrospective analysis of inspection reports from all community pharmacies in the study region (representative voivodeships in Poland) from the period 2002 to 2019 was carried out to assess emergency pharmacist prescribing practices in Poland (first phase of the study). During this time, all prescriptions were paper and not digitalized and could be filled only in an emergency situation. The scope of the study also assessed the preliminary effects of the new pharmaceutical law in Poland that was implemented in April 2020. In the second phase, a retrospective analysis of a database showing pharmacist prescribing habits in the study region from 1 April 2020 to 31 October 2020, when the pharmacy prescription was allowed not only in emergency situations was made. Digitalized data from 2020 was obtained from the e-Health Centre (Centrum e-Zdrowia [CeZ]), a governmental organization under the Ministry of Health responsible for the development of health care information systems in Poland.

The paper data, which were subjected to a first phase of the study were provided by the Regional Pharmaceutical Inspectorate in Bydgoszcz. The researcher, who was legally obliged to act under strict confidentiality terms and disclose only anonymized data, searched the inspectorate’s archives. Every written inspection report from community pharmacies containing lists of issued pharmaceutical prescriptions was included in our study. Documentation from all community pharmacies that were in operation in this area during the study period was analyzed. There were 650 active pharmacies when the collection of the study’s secondary dataset was completed. To obtain a complete picture, we also analyzed the paper records of pharmacies that were no longer in business, but that had operated at least for a short period following the introduction of the term “pharmaceutical prescription” in Polish legislation (i.e., following 1 October 2002). Consequently, data from a total of 842 community pharmacies were included in our analysis. Since the State Pharmaceutical Inspectorate neither collected nor stored data concerning pharmacies and inspections in electronic databases, we had to undertake a labor-intensive examination of paper-based reports stored in folders by pharmacies. The data were gathered from inspection reports issued following comprehensive elective controls conducted by pharmaceutical inspectors who inspected every operational aspect of each pharmacy. Occasionally, inspections’ control of comply by legal regulations concerning pharmaceutical prescriptions was ad hoc. Audited area included a region of Poland with a population of over 2 million inhabitants.

The second stage of the study was based on an analysis of the data on all the pharmacist prescriptions from the public records held by the CeZ. This process involved writing to the CeZ with our research request, in which we were required to demonstrate that there was a significant public interest in the research findings that would result, before we were granted access to the data. We were also required to explain that ours was a scientific study that would generate neutral (objective) results showing trends and how implementation of pharmacist prescribing was working.

### 2.2. Ethical Considerations

The research project received approval from the Independent Bioethics Committee for Scientific Research at Nicolaus Copernicus University of Torun Collegium Medicum in Bydgoszcz (reference number 665/2015). The data acquired was anonymized by a suitable public administrator (Voivodeship Pharmaceutical Inspectorate in Bydgoszcz, CeZ) before sharing for researchers of the content and did not contain any personal data. Only information about the prescribed drugs and the patient’s age was disclosed by both agencies (Voivodeship Pharmaceutical Inspectorate in Bydgoszcz, CeZ), without any possibility to identify a patient. Therefore, no informed consent from the patients was necessary. The study was designed to be a “non-invasive study”. The study was conducted in accordance with the requirements of the Polish Data Protection Act, which implemented Regulation EU 2016/679 of the European Parliament and of the Council of 27 April 2016. According to this act and patients’ rights act, anonymous data (with no possibility to identify the patient) do not need the informed consent from the patient. Data were stored on a Compact Disc (CD) secured with a login password and secured in a strongbox in a locked office.

### 2.3. Data Collection

Analysis of the data gathered from the paper reports allowed us to determine the number of pharmaceutical prescriptions issued, the reasons for dispensing each medicine, and the types of medicine dispensed. The Anatomical Therapeutic Chemical (ATC) Classification System was used to analyze the types of prescribed medicines. Prescriptions did not contain any information about the age of the patient, as this requirement was introduced in 2020. The location for each issuing pharmacy was recorded (i.e., village, city < 100,000, city > 100,000), their location within the town/city (e.g., in the vicinity of a hospital or outpatient clinic, in a shopping center, in a residential area, in the center of the town/city), and their operating hours (i.e., 24-h service, on Sundays and public and bank holidays).

Once we had received CeZ permission to get data from the public database, we were sent all the prescriptions on an encrypted CD. Because of the nature of the data contained in the file and the fact that they included prescriptions, the researchers were required to ensure that the data was kept secure. The data CD received was encrypted and the access code was provided to only one person from the research team. When not in use, the data CD was stored in a key-locked cabinet in a secure room with limited access to authorized personnel only.

### 2.4. Data Analysis

The data were manually digitized in a database using Microsoft Excel (Microsoft Corp., Redmond, WA, USA). Coding and data entry accuracy was checked by a second person from the research team. Statistical analysis was carried out to assess whether the pharmacies’ operating schedule, location, or position within the town/city had any impact on the number of pharmaceutical prescriptions issued or filled. The following statistical tools were used: quantity and percent values, which were also presented as bar graphs; mean and standard deviation calculations; non-parametric Mann–Whitney U test to assess the differences between two populations; non-parametric Spearman’s rank correlation coefficient to assess the relationship between two variables; non-parametric Kruskal–Wallis analysis of variance (ANOVA) rank test to assess the differences when multiple independent groups were compared; post hoc tests for multiple comparisons; and Fisher’s least significant difference (LSD) test. In the second phase of the study, all analyzed variables were nominal using data from the CeZ. Frequency and contingency tables compiled on the basis of both raw data and percentages were used to assess the correlation between the aforementioned. Similar statistical tools were used, including quantity and percent values, which were also presented as bar graphs.

## 3. Results

The first phase of the study analyzed the records of pharmaceutical prescriptions from 842 pharmacies. One hundred and twelve (13.3%) were pharmacies operating in villages and towns with less than 1500 inhabitants, 375 (44.5%) were in towns and cities greater than 1500 and fewer than 100,000 inhabitants, and 355 (42.4%) were pharmacies in cities with a population greater than 100,000. A total of 569 pharmacies (67.6%) operated during standard opening hours Mondays to Saturdays, 17 (2.0%) were open 24 h and seven days a week, 143 pharmacies (17%) operated with extended opening hours, including Sundays, holidays and public holidays, and 113 pharmacies (13.4%) operated periodically on an on-duty basis at times stipulated by local authorities. The nature of the data enabled us to classify each according to their neighborhood characteristics. Three hundred (35.6%) were located in the vicinity of a hospital or an out-patient clinic, 74 (8.8%) operated in shopping centers, 281 (33.4%) were located in residential areas, and 187 (22.2%) were operated in town/city centers. Table 2 presents the quantitative distribution of pharmacies based on their position and the population size of where they were located.

Analysis of pharmacy inspection reports showed that 2189 pharmaceutical prescriptions were issued. The highest incidence rate of prescriptions issued was in cities with a population greater than 100,000. Our data showed that no pharmaceutical prescriptions were issued in pharmacies that were located in villages. With respect to operating hours, the highest rate of issued pharmaceutical prescriptions was in pharmacies open during standard hours from Monday to Saturday (3.04) and those operating periodically on an on-duty basis (1.98); the lowest number of prescriptions issued was in pharmacies open 24 h and seven days a week (0.76 prescriptions). In our data, only three prescriptions were issued at night in pharmacies operating on a 24-h basis. With respect to locations within their town/city, pharmacies in town/city centers and those in residential areas showed the highest index of prescriptions issued (4.14 and 3.48, respectively). The lowest index was observed in pharmacies situated near out-patient clinics or hospitals (1.11) (Table 3).

**Figure 1:**
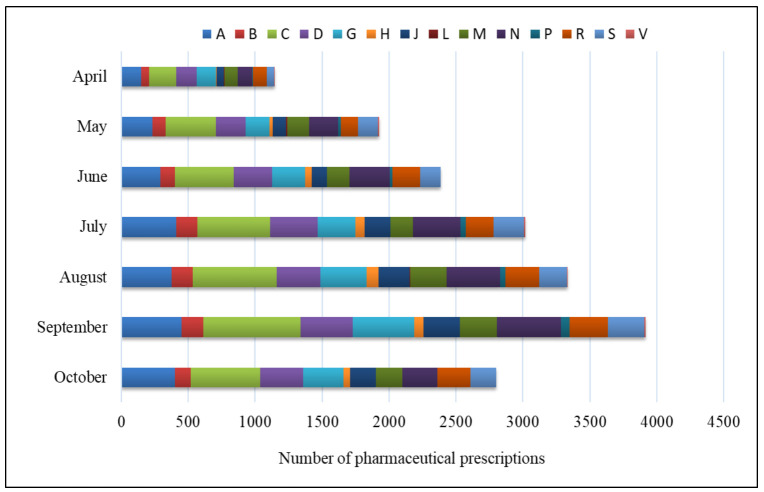
Pharmacist prescribing practices in the Kuyavian-Pomeranian and the types of prescribed medicines according to ATC Classification System from 1 April to 30 October 2020 (data provided by the CeZ); A—Alimentary tract and metabolism, B—Blood and blood forming organs, C—Cardiovascular system, D—Dermatologicals, G—Genito-urinary system and sex hormones, H—Systemic hormonal preparations, excluding sex hormones and insulins, J—Antiinfectives for systemic use, L—Antineoplastic and immunomodulating agents, M—Musculo-skeletal system, N—Nervous system, P—Antiparasitic products, insecticides and repellents, R—Respiratory system, S—Sensory organs, V—Various.

After the introduction of legislation in 2020 for pharmacist prescribing practices in the study region, a steady increase in the number of prescriptions issued by pharmacists was observed; over 1100 prescriptions were issued in April, which increased to almost 3900 in September and consisted of a total of over 18,500 pharmacist prescriptions at evaluated period. A detailed analysis of the number of pharmaceutical prescriptions is presented in Figure 1.

In Table 4, the percentage of medicines prescribed by pharmacist’s including to the ATC Classification System from 1 April to 31 October 2020 is shown. Over a period of seven months, the highest percentage of prescriptions were related to those used for cardiovascular diseases (3436; 18.54%), alimentary tract diseases and metabolism disorders (2316; 12.50%), nervous system (2121; 11.45%), and those used in dermatological diseases (2057; 11.10%).

**Figure 2:**
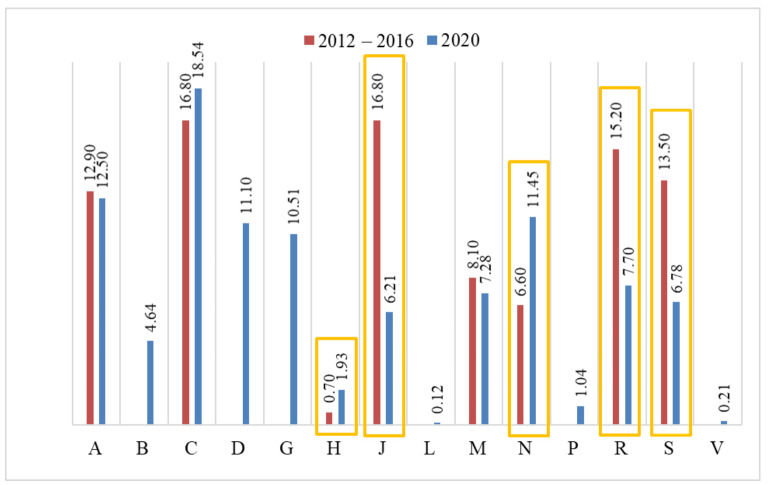
Medicines prescribed by pharmacists according to the ATC Classification System in the Kuyavian-Pomeranian Voivodeship from 2002 to 2016 and from 1 April to 30 October 2020.

Comparing the type of medicines prescribed by pharmacists from 2002–2019 and during 2020 indicated a difference in prescribing practices in ATC groups, including drugs use for systemic hormonal preparations (0.70% vs. 1.93% in 2020), medicines used to treat nervous system disorders (6.60% vs. 11.45% in 2020), anti-infectives medicines for systemic use (16.8% vs. 6.21% in 2020), medicines used in respiratory system diseases (15.20% vs. 7.70% in 2020), and medicines used in sensory organs diseases (13.50% vs. 6.78% in 2020), as shown in Figure 2. Important observations showed anti-infective drugs to have a reduced percentage level in pharmaceutical prescription in this therapeutic group. Additionally, drug prescription by pharmacists of new ATC groups: dermatological group (11.10%), genito-urinary system, and sex-hormones (10.51%) was observed, and no pharmaceutical prescription was found in the first part of the study.

## 4. Discussion

Twenty World Health Organization (WHO) member states (10% of member states) authorize pharmacists and nurses to prescribe medicines, which is widely accepted by both healthcare professionals and patients [10,11,12]. The evolution of pharmacist prescribing practices have differed among these countries. Sweden adopted the idea of “repeat prescribing” [13], while Canada and the United Kingdom (UK) adopted so-called “independent prescribing” [14,15,16,17,18]. One reason many developing and developed countries introduced pharmacist prescribing was to give patients better access to healthcare services, including access to medicines. Studies also indicate the importance pharmacist prescribing after-hours and in emergency situations [19,20]. Published studies on emergency prescribing practices are scarce. One UK study [4] analyzed the emergency dispensing processes of prescription-only medicines by pharmacists. The four-week, four-stage study took place in 22 community pharmacies with varying ownership arrangements, gathered data from patients, pharmacists, and general physicians. Eight pharmacies were in the vicinity of a healthcare facility, nine in shopping centers, three in town/city centers, and two in other parts of the town/city. Fourteen pharmacies operated Monday to Friday, five from Monday to Saturday, and three included Sunday trading. A total of 526 medicinal products were dispensed to 450 patients (in 90% of cases, the prescription was for a single preparation). The need for pharmacist intervention increased on weekends and public holidays was observed. Overall, most dispensing occurred on Mondays or Fridays. Fridays accounted for nearly 25% of all cases. Most dispensing was for elderly patients (137 events for patients aged 60–74, 116 for those aged 45–59, and 94 for those older than 75 years). Predominantly, patients sought medicines to continue their current therapy, a similar finding observed in our study. The medicines that were most commonly dispensed were for cardiovascular (32%), respiratory (13%), endocrine (12%), and gastrointestinal diseases (11%). The UK study indicates that demand for emergency and repeat prescribing emphasizes the important role of the pharmacist and community pharmacies in the healthcare system. An English study showed that patients commonly sought a recurrent need for medicines related to cardiovascular, endocrine, and gastrointestinal diseases [21]. The demand for medicines used to treat respiratory diseases was half of that found in the Morecroft et al. study [4]. In a study based on 401 pharmacists by George et al. that evaluated cases of supplementary prescribing, prescribed medicines were used mostly for cardiovascular diseases [17]. One American study indicated that 75% of pharmacists only fill emergency prescriptions several times a month and that they may dispense antibiotics, inhaled medications, antidiabetic medicines, and drugs used for nausea and vomiting [22]. Our own data analysis indicated that the most common prescriptions were for cardiovascular medicines and anti-infectives. Our study showed that most cases of pharmacist prescribing took place during normal business hours, similar to findings in Morecroft et al. [4].

Pharmacists who prescribe medicines for themselves or their family members when a long-term medicine was running low is a common practice and was granted legal status in the 2020 Law that included reimbursable and non-reimbursable medicines. Though providing healthcare services to oneself and/or family members is generally considered inappropriate and may be regarded as a conflict of interest, issuing a prescription to any person in an emergency situation, or when another appropriate health professional is not readily available, are both permitted.

In addition to peer-reviewed scientific studies, data on pharmacist prescribing practices may be gathered from official health sector reports such as the Prescription Analysis and Cost (PACT) reports in the UK. In the report summarizing the first period after the introduction of emergency prescribing in the UK (2004–2006), the data indicate that pharmacist prescribing practices initially remained low, but then increased from 2706 in 2004 to 31,052 by 2006; however, these values represent a small proportion (0.004%) of all medicines prescribed in UK healthcare facilities. The medicines most commonly prescribed by pharmacists were those used for chronic diseases, including cardiovascular (mainly acetylcholinesterase converting enzyme inhibitors, diuretics, nitrates, calcium channel blockers, antianginal medicines, and lipid level regulating drugs), nervous system therapy (pain medication, anxiolytics and hypnotics), respiratory conditions (bronchodilatators, mainly corticosteroids), endocrine disorders (for diabetes in more than 50% of cases), and gastrointestinal diseases. In monetary terms, emergency prescribing practices increased from GBP 25,348 in 2004 to GBP 278,634 in 2006 [23]. Studies have shown that oftentimes, when patients are unable to access emergency supply services, they stop taking their medication. In a UK study of 227 community pharmacies, a total of 2485 patients needed emergency dispensing and most went to the pharmacy on Saturdays and national holidays. The elderly were heavily represented in these data. Of 3226 dispensing cases, 439 were classified as high-risk events. Patients’ easy access to the emergency services increased their willingness to contact the community pharmacy in the future for medication-related issues [24]. Studies have reported medication-related non-adherence as a frequent reason for hospital admissions [25]. Not having access to medications used in long-term therapy may pose a significant risk to the patient.

For the first time, data on pharmacist prescribing practices in study region after the introduction of legislation effecting pharmacist prescribing practices showed a steady increase in the number of prescriptions issued by pharmacists from April to October 2020. This confirms the impact of recent legislative changes and how pharmacists can help with the prescription of medicines to address health needs. Moreover its demonstrate the effective role of researchers (science advocacy) in helping preparation and validation of new regulatory solutions [26].

This study has a few limitations to note. Our study is a descriptive study, so the subject of the research was not an assessment of the adequacy of the prescriptions and economic evaluation of the costs of this practice. The identification of factors related to patient characteristics was not carried out by the investigators. Pre- and pending pandemic differences in the prescribed drugs was identified only according to ATC groups. The results presented in the figures illustrated the observing changes adequately to pharmacy law transformation and changes of pharmacist prescribing practices in Poland.

## 5. Conclusions

Polish pharmacists most commonly issue prescriptions for medicines used for cardiovascular diseases, alimentary tract and metabolism, nervous system, or for those used in respiratory system diseases. This indicates that patients regard pharmacies as important healthcare facilities. The observed decreased trend in the pharmaceutical prescription of anti-invectives drugs is an expected phenomena, especially in the case of increasing antibiotics resistance and the lack in pharmacies of rapid diagnostic tests to identification of infection cause. Our study shows that retaining pharmacist prescribing practices in the Polish healthcare system is justified. The application of these kind of results help advocate for science and help demonstrate the role that research has contributed to new legislation in Poland. This indicates that such studies are required in shaping policy and legislation regarding pharmacist dispensing practices, as evidenced by the analysis of pharmacist prescribing practices following the legislative amendment.

## Figures and Tables

**Table 1:** Practice differences for issuing prescriptions in Poland before and after the COVID-19 epidemic was declared and law regulatory changes [5].

Pharmacist Prescribing Rules	Before 31st March 2020	After 1st April 2020
Prescription type	Only the paper version was technically possible	The electronic prescription is preferred and made available. The paper version only in exceptional cases
Reasons for issuing a prescription	Immediate health risk	Health risk
Amount of medicine	The smallest, single registered packet of the given medicine	In the case of electronic prescriptions, the amount of medicine required for 180 days of therapy for the given dose
Types of medicines	Prescription-only medicines except narcotics and psychotropic medication	Prescription-only medicines except narcotics and psychotropic medication
Information needed on the prescription	The same as on the prescription issued by a physician, and the reason for dispensing the medicine	The same as on the prescription issued by a physician and the reason for dispensing the medicine
Reimbursement	0%	0%, excluding prescriptions issued for the pharmacist himself/herself or his/her family members

**Table 2:** The distribution of the studied pharmacies based on their location and position within the town/city.

Location	Rural Area		City < 100,000		City > 100,000
Position	*n*	%	*n*	%	*n*
Neighborhood of out-patient clinic or hospital	59	52.7	134	35.7	107
Shopping center	2	1.8	35	9.3	37
Residential area	43	38.4	85	22.7	153
Town/city center	8	7.1	121	32.3	58
Total	112	100.0	375	100.0	355

**Table 3:** Statistical analysis of the number of pharmaceutical prescriptions based on pharmacy location.

Pharmacy Location	*n*	Mean	SD	CI −95.0%	CI +95.0%	Minimum	Maximum
Neighborhood of out-patient clinic or hospital	300	1.11	13.421	−0.42	2.63	0.0	218.0
Shopping center	74	1.42	7.503	−0.32	3.16	0.0	53.0
Residential area	281	3.48	30.094	−0.05	7.01	0.0	431.0
Town/city center	187	4.14	30.816	−0.31	8.58	0.0	371.0

**Table 4:** Prescribed drugs by pharmacists according to the ATC Classification System in the analyzed region from 1st April to 30th October 2020.

ATC Code	Total Pharmacist Prescribing 1 April–30 October	Percentage of Pharmacist’s Prescribing in the Month							∑ [%]
		April	May	June	July	August	September	October	
A	2316	0.80	1.27	1.59	2.23	2.03	2.43	2.16	12.50
B	859	0.32	0.51	0.58	0.83	0.86	0.87	0.65	4.64
C	3436	1.10	2.02	2.36	2.95	3.37	3.93	2.81	18.54
D	2057	0.82	1.21	1.55	1.93	1.77	2.09	1.73	11.10
G	1948	0.78	0.94	1.33	1.51	1.86	2.48	1.62	10.51
H	357	0.02	0.13	0.26	0.38	0.47	0.39	0.27	1.93
J	1151	0.30	0.54	0.62	1.02	1.25	1.46	1.03	6.21
L	22	0.02	0.05	0.00	0.00	0.04	0.00	0.00	0.12
M	1348	0.52	0.89	0.91	0.91	1.47	1.51	1.08	7.28
N	2121	0.60	1.16	1.62	1.93	2.16	2.57	1.40	11.45
P	193	0.02	0.11	0.13	0.23	0.22	0.34	0.00	1.04
R	1427	0.56	0.70	1.10	1.10	1.34	1.55	1.35	7.70
S	1256	0.30	0.78	0.84	1.25	1.12	1.46	1.03	6.78
V	38	0.02	0.05	0.00	0.04	0.04	0.05	0.00	0.21
∑	18,529	6.20	10.39	12.89	16.28	18.00	21.13	15.11	100.00

A—Alimentary tract and metabolism, B—Blood and blood forming organs, C—Cardiovascular system, D—Dermatologicals, G—Genito-urinary system and sex hormones, H—Systemic hormonal preparations, excluding sex hormones and insulins, J—Antiinfectives for systemic use, L—Antineoplastic and immunomodulating agents, M—Musculo-skeletal system, N—Nervous system, P—Antiparasitic products, insecticides and repellents, R—Respiratory system, S—Sensory organs, V—Various.

## Data Availability

There are legal restrictions on sharing a de-identified data set, as specified by the Medical University of Gdańsk’s inner regulations dedicated to employees. However, all surveys data is available upon request to researchers who meet the criteria for access to confidential data. Requests may be sent to the corresponding author.

## References

[1] Zimmermann A., Cieplikiewicz E., Wąż P., Gaworska-Krzemińska A., Olczyk P. (2020). The implementation process of nurse prescribing in Poland—A descriptive study. Int. J. Env. Res. Public Health.

[2] Hibbert D., Rees J.A., Smith I. (2000). Ethical awareness of community pharmacists. Int. J. Pharm. Pract..

[3] Yuksel N., Eberhart G., Bungard T.J. (2008). Prescribing by pharmacists in Alberta. Am. J. Health Syst. Pharm..

[4] Morecroft C.W., Mackridge A.J., Stokes E.C., Gray N.J., Wilson S.E., Ashcroft D.M., Mensah N., Pickup G.B. (2015). Emergency supply of prescription-only medicines to patients by community pharmacists: A mixed methods evaluation incorporating patient, pharmacist and GP perspectives. BMJ Open.

[5] Pharmaceutical Law of 6 September 2001 (JL No. 126, item 1381) Consolidated Text of 15 March 2019 (JL item 499) and Consolidated Text of 28 May 2021 (JL item 974). http://isap.sejm.gov.pl/isap.nsf/DocDetails.xsp?id=wdu20011261381.

[6] Kim J.J., Tian A.H., Pham L., Nakhla N., Houle S.K., Wong W.W., Alsabbagh M.W. (2021). Economic evaluation of pharmacists prescribing for minor ailments in Ontario, Canada: A cost-minimization analysis. Int. J. Pharm. Prac..

[7] Mossialos E., Courtin E., Naci H., Benrimoj S., Bouvy M., Farris K., Noyce P., Sketris I. (2015). From “retailers” to health care providers: Transforming the role of community pharmacists in chronic disease management. Health Policy.

[8] Nachtigal P., Šimůnek T., Atkinson J. (2017). Pharmacy Practice and Education in the Czech Republic. Pharmacy.

[9] Soares I.B., Imfeld-Isenegger T.L., Makovec U.N., Horvat N., Kos M., Arnet I., Hersberger K.E., Costa F.A. (2020). A survey to assess the availability, implementation rate and remuneration of pharmacist-led cognitive services throughout Europe. Res. Soc. Adm. Pharm..

[10] Weiss M.C., Sutton J. (2009). The changing nature of prescribing: Pharmacists as prescribers and challenges to medical dominance. Sociol. Health Illness.

[11] Latter S., Blenkinsopp A., Smith A., Chapman S., Tinelli M., Gerard K., Little P., Celino N., Granby T., Nicholls P. (2011). Evaluation of Nurse and Pharmacist Independent Prescribing.

[12] Bhanbhro S., Drennan V.M., Grant R., Harris R. (2011). Assessing the contribution of prescribing in primary care by nurses and professionals allied to medicine: A systematic review of literature. BMC Health Serv. Res..

[13] Andersson K., Melander A., Svensson C., Lind O., Nilsson J.L.G. (2005). Repeat prescriptions: Refill adherence in relation to patient and prescriber characteristics, reimbursement level and type of medication. Eur. J. Public Health.

[14] Riley R., Weiss M.C., Platt J., Taylor G., Horrocks S., Taylor A. (2013). A comparison of GP, pharmacist and nurse prescriber responses to patients’ emotional cues and concerns in primary care consultations. Patient Educ. Couns..

[15] Famiyeh I.M., MacKeigan L., Thompson A., Kuluski K., McCarthy L.M. (2019). Exploring pharmacy service users’ support for and willingness to use community pharmacist prescribing services. Res. Soc. Adm. Pharm..

[16] Hoti K., Hughes J., Sunderland B. (2013). Expanded prescribing: A comparison of the views of Australian hospital and community pharmacists. Int. J. Clin. Pharm..

[17] George J., Pfleger D., McCaig D., Bond C., Stewart D. (2006). Independent prescribing by pharmacists: A study of the awareness, views and attitudes of Scottish community pharmacists. Pharm. World Sci..

[18] Auta A., Strickland-Hodge B., Maz J. (2016). Stakeholders’ views on granting prescribing authority to pharmacists in Nigeria: A qualitative study. Int. J. Clin. Pharm..

[19] Smith J., Picton C., Dayan M. (2013). Now or never: Shaping pharmacy for the future. The Report of the Commission on Future Models of Care Delivered through Pharmacy.

[20] Law M.R., Morgan S.G., Majumdar S.R., Lynd L.D., Marra C.A. (2010). Effects of prescription adaptation by pharmacists. BMC Health Serv. Res..

[21] O’Neill R., Rowley E., Smith F. (2002). The emergency supply of prescription-only medicines: A survey of requests to community pharmacists and their views on the procedures. Int. J. Pharm. Pract..

[22] Shepherd M.D. (2013). Examination of why some community pharmacists do not provide 72-hour emergency prescription drugs to Medicaid patients when prior authorization is not available. J. Manag. Care Spec. Pharm..

[23] Guillaume L., Cooper R., Avery A., Mitchell S., Ward P., Anderson C., Bissell P., Hutchinson A., James V., Lymn J. (2008). Supplementary prescribing by community pharmacists: An analysis of PACT data, 2004–2006. J. Clin. Pharm. Ther..

[24] Nazar H., Nazar Z., Simpson J., Yeung A., Whittlesea C. (2016). Summative service and stakeholder evaluation of an NHS-funded community Pharmacy Emergency Repeat Medication Supply Service (PERMSS). BMJ Open.

[25] Howard R.L., Avery A.J., Howard P.D., Partridge M. (2003). Investigation into the reasons for preventable drug related admissions to a medical admissions unit: Observational study. Qual. Saf. Health Care.

[26] Runkle D., Frankel M.S. (2012). Advocacy in science. Summary of Workshop Convened by the American Association for the Advancement of Science.

